# Supplementary material for: Nanoscale binding site localization by molecular distance estimation on native cell surfaces using topological image averaging
Source: eLife. 2022 Feb 24;11:e64709. doi: 10.7554/eLife.64709 (PMC8871386; doi:10.7554/eLife.64709)
Supplement: Transparent reporting form [file elife-64709-transrepform1.docx]

***eLife’s* transparent reporting form**

We encourage authors to provide detailed information *within their submission* to facilitate the interpretation and replication of experiments. Authors can upload supporting documentation to indicate the use of appropriate reporting guidelines for health-related research (see [EQUATOR Network](http://www.equator-network.org/)), life science research (see the [BioSharing Information Resource](https://biosharing.org/)), or the [ARRIVE guidelines](http://www.plosbiology.org/article/info:doi/10.1371/journal.pbio.1000412) for reporting work involving animal research. Where applicable, authors should refer to any relevant reporting standards documents in this form.

If you have any questions, please consult our Journal Policies and/or contact us: [editorial@elifesciences.org](mailto:editorial@elifesciences.org).

**Sample-size estimation**

- You should state whether an appropriate sample size was computed when the study was being designed
- You should state the statistical method of sample size computation and any required assumptions
- If no explicit power analysis was used, you should describe how you decided what sample (replicate) size (number) to use

Please outline where this information can be found within the submission (e.g., sections or figure legends), or explain why this information doesn’t apply to your submission:

Since the paper describes a novel measurement method a certain significance was not pursued. Instead, the aim was to describe the statistical properties of the method and distribution of the measurements (see **Statistical reporting** below).

**Replicates**

- You should report how often each experiment was performed
- You should include a definition of biological versus technical replication
- The data obtained should be provided and sufficient information should be provided to indicate the number of independent biological and/or technical replicates
- If you encountered any outliers, you should describe how these were handled
- Criteria for exclusion/inclusion of data should be clearly stated
- High-throughput sequence data should be uploaded before submission, with a private link for reviewers provided (these are available from both GEO and ArrayExpress)

Please outline where this information can be found within the submission (e.g., sections or figure legends), or explain why this information doesn’t apply to your submission:

The number of cells imaged in each experiment is stated in each figure caption (Fig 3,4 and 5, n between 25 and 48). For all experiments, each cell is imaged repeatedly 15-20 times to acquire a high averaged signal as stated in the methods section (SIM acquisition).

**Statistical reporting**

- Statistical analysis methods should be described and justified
- Raw data should be presented in figures whenever informative to do so (typically when N per group is less than 10)
- For each experiment, you should identify the statistical tests used, exact values of N, definitions of center, methods of multiple test correction, and dispersion and precision measures (e.g., mean, median, SD, SEM, confidence intervals; and, for the major substantive results, a measure of effect size (e.g., Pearson's r, Cohen's d)
- Report exact p-values wherever possible alongside the summary statistics and 95% confidence intervals. These should be reported for all key questions and not only when the p-value is less than 0.05.

Please outline where this information can be found within the submission (e.g., sections or figure legends), or explain why this information doesn’t apply to your submission:

The paper outlines a statistical averaging method as described in Fig. 1 and section “Principles of site localization method”. Each site localisation value is calculated as the mean of all experimental or simulated replicates and the precision of the measurements is given as both SD and IQR depending on the reported average as stated in the method (Site localisation analysis). Median(IQR) and/or Mean +/- SD is given in each caption. The datasets are illustrated as violin plots with median and IQR indicated as a bar in Fig 3, 4, 5, F3S1, F3S2, F4S2 and F5S1. In Figure 3e the datasets are illustrated in a scatter plot with median value marked and IQR as error bars.

The reported measurement accuracy is assessed by estimating the 95% confidence interval of the mean of a number of replicates computed by case resampling percentile bootstrap method (Fig 2, F4S1).

(For large datasets, or papers with a very large number of statistical tests, you may upload a single table file with tests, Ns, etc., with reference to sections in the manuscript.)

**Group allocation**

- Indicate how samples were allocated into experimental groups (in the case of clinical studies, please specify allocation to treatment method); if randomization was used, please also state if restricted randomization was applied
- Indicate if masking was used during group allocation, data collection and/or data analysis

Please outline where this information can be found within the submission (e.g., sections or figure legends), or explain why this information doesn’t apply to your submission:

Not applicable. The data was collected solely on the basis of successful staining. As the resolution of the raw data is too low for differentiation of binding site location, the microscope operator can not bias the data collection. All analysis was performed automatically using the provided code.

**Additional data files (“source data”)**

- We encourage you to upload relevant additional data files, such as numerical data that are represented as a graph in a figure, or as a summary table
- Where provided, these should be in the most useful format, and they can be uploaded as “Source data” files linked to a main figure or table
- Include model definition files including the full list of parameters used
- Include code used for data analysis (e.g., R, MatLab)
- Avoid stating that data files are “available upon request”

Please indicate the figures or tables for which source data files have been provided:

Complete source code provided on Github with link in discussion. Analysis described in methods section.
